# The patterns and timing of recurrence after curative resection for gastric cancer in China

**DOI:** 10.1186/s12957-016-1042-y

**Published:** 2016-12-08

**Authors:** Dan Liu, Ming Lu, Jian Li, Zuyao Yang, Qi Feng, Menglong Zhou, Zhen Zhang, Lin Shen

**Affiliations:** 1 Key Laboratory of Carcinogenesis and Translational Research (Ministry of Education), Department of Gastrointestinal Oncology, Peking University Cancer Hospital and Institute, No. 52, Fucheng Road, Haidian District, Beijing, 100142 China; 2 Division of Epidemiology, The Jockey Club School of Public Health and Primary Care, The Chinese University of Hong Kong, Hong Kong, 999077 Hong Kong China; 3 Department of Radiation Oncology, Shanghai Cancer Center, Fudan University, Shanghai, 200032 China

**Keywords:** Gastric cancer, Curative resection, Postoperative recurrence, Disease-free survival, Overall survival

## Abstract

**Background:**

The recurrence of gastric cancer after curative resection had adverse effects on patients’ survival. The treatment presence varied from different countries. The aims of this study were to understand the recurrence incidence, patterns, and timing and to explore the risk factors in China.

**Methods:**

One thousand three hundred four patients who undergoing curative resection from more than 100 hospitals between January 1st 1986 and September 1st 2013, were surveyed in detail. Clinical pathological factors were examined as potential risk factors of each recurrence pattern using univariate and multivariate analyses. Recurrence timing was also analyzed based on disease-free survival.

**Results:**

Among 1304 gastric cancer patients, 793 patients (60.8%) experienced recurrence and 554 patients (42.5%) experienced recurrence within 2 years after operation. The median disease-free survival was 29.00 months (interquartile range [IQR] 12.07, 147.23). Receiving operation in general hospitals was one of independent risk factors of local-regional recurrence (OR = 1.724, 95% CI 1.312 to 2.265) and distant metastasis (OR = 1.496, 95% CI 1.164 to 1.940). Patients would suffer lower risk of distant metastasis if they received no more than 3 cycles adjuvant chemotherapy (OR = 0.640, 95% CI 0.433 to 0.943). Adjuvant radiotherapy could reduce the risk of recurrence (OR 0.259, 95% CI 0.100 to 0.670), especially distant metastasis (OR = 0.260, 95% CI 0.083 to 0.816).

**Conclusions:**

More than 60% patients experienced recurrence after curative resection for gastric cancer, especially within 2 years after surgery. Risk factors were clarified between various recurrence patterns. Advanced gastric cancer and undergoing operation in general hospitals contributed to increased recurrence risk and worse survival. Enough number of lymph nodes harvest and standard D2 lymphadenectomy could reduce recurrence. Chinese patients would benefit from adjuvant chemotherapy and radiotherapy.

## Background

Gastric cancer is the fourth common malignant tumor in the world, and China is one of countries with high gastric cancer incidence [1, 2]. So far, curative resection has been considered as the only way to cure gastric cancer. Recurrence after curative resection contributes to the limited survival of patients. Recurrence patterns generally include local-regional recurrence, distant or hematogenous metastasis, and peritoneum implanting. Recurrence patterns have related to adjuvant treatment modes. For example, in America, local-regional recurrence and hematogenous metastasis were fairly common, and patients could benefit from adjuvant radio-chemotherapy [3–5]. While, in Japan and South Korea, where distant metastasis and peritoneum implanting were regular, patients could benefit from adjuvant chemotherapy, rather than adjuvant radio-chemotherapy [6–9]. Moreover, recurrence timing could provide information of postoperative follow-up, in order to find recurrence timely. In China, several small sample single-center studies had reported the recurrence patterns and relative risk factors [10–12], but little large sample and multiple centers analysis had reported recurrence timing and survival. And the level of gastric cancer treatment varied from different hospitals, sites, and times. So, that exploring the actual recurrence patterns and timing was necessary. It was the first large sample of multiple-center retrospective analysis for recurrence patterns and timing after curative gastric cancer resection in China. We specifically analyzed the relationship between surgical hospitals and recurrence. It would provide more information for clinicians in choosing individual treatment schedules and predicting prognosis.

## Methods

### Patients

**Fig. 1:**
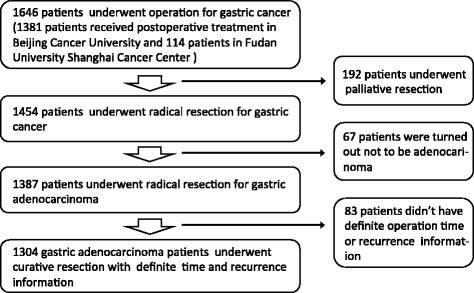
The screening progress for analysis patients

There were 1646 patients, who underwent operation for gastric malignant tumors between January 1st 1986 and September 1st 2013, in more than 100 Chinese hospitals, screened for this analysis. All of them finally received postoperative treatment in Gastrointestinal Tumor Department of Beijing Cancer Hospital and Radiotherapy Department of Fudan University Shanghai Cancer Center. Three hundred forty-two patients were excluded for the following reasons: (1) 192 patients underwent palliative tumor resection (including both primary and non-local regional metastasis lesions totally resected) or experienced microscopically/visible positive (R1/R2) margin status, (2) 67 patients whose postoperative histological examinations turned out not to be gastric adenocarcinoma, and (3) 83 patients experienced death whose recurrence time and patterns were unclear. Finally, 1304 gastric adenocarcinoma patients were included in this study for recurrence patterns and timing after curative resection (Fig. 1).

### Data collection

All patients’ clinical pathological characteristics, including age, sex, tumor location, surgical hospitals (general and specialist hospitals), depth of tumor invasion, number of positive lymph nodes, extent of lymphadenectomy, histological type, neo-adjuvant treatment, adjuvant treatment, and recurrence and survival information were retrospectively reviewed based on operative notes and medical records.

### Pathological identification

Cardia and fundus tumors were identified as gastroesophageal junction (GEJ) adenocarcinoma. Tumors located in the rest sites of gastric were identified as non-gastroesophageal junction (non-GEJ) adenocarcinoma. The lymphadenectomy extent was defined according to the 2010 Japanese gastric cancer treatment guidelines [13]. The tumors’ stages were classified based on the postoperative tissues, according to the 7th edition American Joint Committee on Cancer (AJCC) staging system of gastric adenocarcinoma. Histological classification was in accordance with the World Health Organization (WHO) classification of tumors of the digestive system [14]. Well-differentiated tumors included highly and moderately differentiated papillary carcinoma. And poorly differentiated included low differentiated papillary carcinoma, mucinous adenocarcinoma, and hepatoid adenocarcinoma. Recurrence was defined as biopsy and imaging highly suspicious of recurrence. Recurrence patterns included local-regional recurrence (gastric or nodal), distant metastasis (organs and distant lymph nodes), and peritoneum implanting (peritoneum nodules, ascites, and Krukenberg tumors).

### Postoperative follow-up

All patients were followed from the date of surgery to death or emigration. The last follow-up of all recurrence and survival information was January 7th 2015. Recurrence and survival data were obtained from patients’ medical records and telephone follow-up.

The recurrence free survival (RFS) was defined as the time from surgery to recurrence or death of any other causes. The overall survival (OS) was defined as the time from operation to death. The recurrence occurred within 2 years after surgery was defined as early recurrence. Recurrence patterns were classified based on the site of the first recurrence. The recurrence inspected within 3 months after the first recurrence was regarded as synchronous recurrence.

### Statistical analysis

Fifteen clinical relative characteristics were examined as potential risk factors of recurrence. Differences between two groups were assessed by the chi-square or Fisher exact tests. The association of clinical pathological factors with the extent of recurrence was assessed using logistic models. Back-Wald method of multivariate analysis model was used to avoid possible interaction factors for recurrence patterns. Survival curves were analyzed by Kaplan-Meier method and compared by the log-rank test. All analyses were carried out by SPSS version 22.0. The prognostic powers of covariates were recorded by odds ratios (ORs) and 95% confidence internals (CIs). All *p* values <0.05 were considered statistically significant.

## Results

### Clinical pathological characteristics

Among the 1304 analyzed patients, the median age was 58.0 years old (IQR 48.0, 65.0). There were 981 males (75.2%) and 323 females (24.8%), with a male-to-female ratio of nearly 3:1. The most general tumor location was the antrum (*n* = 437, 33.5%) and cardiac (*n* = 387, 29.7%). Most patients (*n* = 1106, 84.8%) underwent subtotal gastrectomy. The median number of total lymph nodes dissection was 16 (IQR 10.24). And 697 (53.5%) patients had lymph nodes dissection no less than 15. Patients had more lymph nodes harvest in specialist hospitals than in general hospitals (median number of lymph nodes harvest 19 [IQR 13.27] vs. 13 [IQR 8.22]). And the specialist hospitals had higher D2 lymphadenectomy rate than general hospitals (63.0 vs. 39.0%, *p* < 0.001). The median number of positive lymph nodes harvest was 3 (IQR 0.7). A majority of patients (*n* = 1195, 91.6%) suffered advanced gastric cancer. Only 63 patients (4.8%) experienced neo-adjuvant chemotherapy, and nobody received neo-adjuvant radiotherapy. A large subset of patients (*n* = 1020, 78.2%) received adjuvant chemotherapy. Nine hundred seventy patients (74.4%) received fluorouracil-based chemotherapy, and 44 patients (3.4%) received other non-fluorouracil-based chemotherapy. Almost half of patients (*n* = 647, 49.6%) received adjuvant chemotherapy over 3 cycles. Only 26 patients (2.0%) had adjuvant radiotherapy (Table 1).

**Table 1:** The clinical pathological characteristics of patents

Clinical features	No. of patients (*n* = 1304)
Age, median year, IQR	58.0 years(48.0, 65.0)
Sex, *n*(%)	
Male	981(75.2%)
Female	323(24.8%)
Surgical hospitals, *n*(%)	
General hospital	695(53.3%)
Specialist hospital	600(46.0%)
Unknown	9(0.7%)
Tumor location, *n*(%)	
Gastroesophageal junction	435(33.4%)
Non-gastroesophageal junction	865(66.3%)
Unknown	4(0.3%)
Histological type, *n*(%)	
Well-differentiated tumors	301(23.1%)
Poorly differentiated tumors	842(64.6%)
Signet ring cell cancer	142(10.9%)
Unknown	19(1.5%)
Surgical approach, *n*(%)	
Proximal gastrectomy	384(29.4%)
Distal gastrectomy	722(55.4%)
Total gastrectomy	197(15.1%)
Unknown	1(0.1%)
Lymphadenectomy type	
D2 lymphadenectomy	627(48.1%)
D0/D1 lymphadenectomy	621(47.6%)
Unknown	56(4.3%)
T stage, *n*(%)	
T1	100(7.7%)
T2	190(14.6%)
T3	586(44.9%)
T4a	367(28.1%)
T4b	52(4.0%)
Unknown	9(0.7%)
N stage, *n*(%)	
N0	326(25.0%)
N1	265(20.3%)
N2	319(24.5%)
N3	373(28.6%)
Unknown	21(1.6%)
Number of LN dissection	
<15	561(43.0%)
≥15	697(53.5%)
Unknown	46(3.5%)
AJCC stage, *n*(%)	
IA	66(5.1%)
IB	91(7.0%)
IIA	174(13.3%)
IIB	218(16.7%)
IIIA	262(20.1%)
IIIB	302(23.2%)
IIIC	164(12.6%)
Unknown	27(2.1%)
Neo-adjuvant chemotherapy	
Yes	63(4.8%)
No	1177(90.3%)
Unknown	64(4.9%)
Adjuvant chemotherapy regimen	
No adjuvant chemotherapy	188(14.4%)
Fluorouracil-based regimens	970(74.4%)
Other regimens	44(3.4%)
Unknown	102(7.8%)
Adjuvant chemotherapy cycles	
No adjuvant chemotherapy	188(14.4%)
≤3 cycles	370(28.4%)
>3 cycles	647(49.6%)
Unknown	99(7.6%)
Adjuvant chemotherapy	
No	188(14.4%)
Yes	1020(78.2%)
Unknown	96(7.4%)
Adjuvant radiotherapy	
Yes	26(2.0%)
No	1209(92.7%)
Unknown	69(5.3%)

### Recurrence patterns and recurrence-related risk factors

#### Patterns of recurrence and risk factors associated with postoperative recurrence

**Fig. 2:**
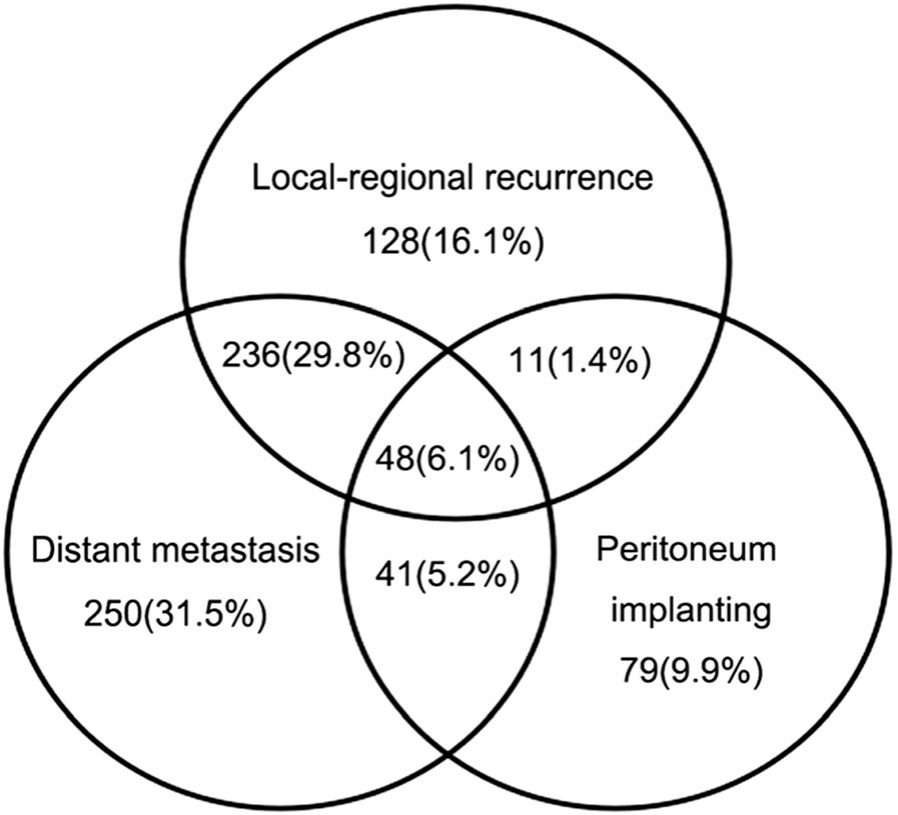
The recurrence patterns of patients with curative gastrectomy

The median follow-up was 56.58 months (IQR 30.45, 105.40), and 116 patients (8.7%) were lost during the follow-up. Seven hundred ninety-three patients (60.8%) suffered recurrence. And there were 423 patients (32.4%) with local-regional recurrence, 575 patients (44.3%) with distant metastasis, and 179 patients (13.7%) suffering peritoneum implanting (Fig. 2). Four hundred fifty-seven patients (57.5%) experienced single pattern recurrence, while 336 patients (42.5%) experienced multiple pattern recurrence. The distant lymph node metastasis was more general than distant organ metastasis (*n* = 392, 49.4 vs. *n* = 294, 37.0%). The general organs of distant metastasis included liver (*n* = 154), lung (*n* = 40), bone (*n* = 38), pleura (*n* = 25), and subcutaneous nodule metastasis (*n* = 23).

Based on this database, several clinical pathological factors were independent risk factors of recurrence in any site (Table 2). Specially, patients who underwent radical surgery in general hospitals (OR 1.632, 95% CI 1.238 to 2.151) and suffered signet ring cell cancer (OR 1.881, 95% CI 1.108 to 3.193) were more likely to experience recurrence. Patients receiving adjuvant radiotherapy (OR 0.259, 95% CI 0.100 to 0.670) contributed to lower recurrence risk.

**Table 2:** The univariate and multivariate analysis of postoperative recurrence

Clinical features	Univariate analysis			Multivariate analysis		
	OR	95% CI	*p* value	OR	95% CI	*p* value
Age						
≦65						
>65	1.178	0.916–1.514	0.201	NA	NA	NA
Sex						
Male						
Female	0.837	0.648–1.080	0.171	NA	NA	NA
Hospital						
Specialist hospitals						
General hospitals	1.915	1.529–2.398	<0.001	1.632	1.238–2.151	0.001
Tumor location						
Non-GEJ						
GEJ	1.683	1.319–2.146	<0.001	1.431	1.066–1.922	0.017
AJCC stage						
I–II						
III	2.608	2.070–3.286	<0.001	NA	NA	NA
T stage						
T1–2						
T3–4	2.861	2.188–3.741	<0.001	2.332	1.675–3.245	<0.001
N stage						
N0						
N1–3	3.397	2.617–4.409	<0.001	2.085	1.481–2.936	<0.001
Positive LN ratio						
≦0.33						
>0.33	3.577	2.759–4.638	<0.001	2.283	1.656–3.145	<0.001
No. of LN dissection						
<15						
≧15	0.502	0.397–0.635	<0.001	NA	NA	NA
Histological type						
Well-differentiated tumors						
Poorly differentiated tumors	0.845	0.645–1.107	0.222	0.792	0.570–1.099	0.162
Signet ring cell cancer	2.066	1.307–3.265	0.002	1.881	1.108–3.193	0.019
Lymphadenectomy extent						
D2						
D0/D1	1.923	1.525–2.424	<0.001	2.361	1.771–3.147	<0.001
Neo-adjuvant chemotherapy						
Yes						
No	1.260	0.756–2.100	0.374	NA	NA	NA
Adjuvant chemotherapy						
No						
Yes	0.978	0.710–1.347	0.892	NA	NA	NA
Adjuvant chemotherapy regimen						
No						
Fluorouracil-based regimens	0.947	0.687–1.306	0.740	NA	NA	NA
Other regimens	2.414	1.096-5.315	0.029	NA	NA	NA
Adjuvant chemotherapy cycles						
No						
≦3 cycles	0.754	0.527–1.080	0.123	NA	NA	NA
>3 cycles	1.156	0.827–1.617	0.396	NA	NA	NA
Adjuvant radiotherapy						
Yes						
No	2.152	0.980–4.725	0.056	3.868	1.493–10.022	0.005

#### Risk factors of each recurrence pattern

In exploring risk factors of recurrence patterns, different clinical pathological factors contributed to specific recurrence patterns (Table 3). Receiving operation general hospitals independently risk factors of local-regional recurrence (OR = 1.724, 95% CI 1.312 to 2.265) and distant metastasis (OR = 1.496, 95% CI 1.164 to 1.940). D0/D1 lymphadenectomy increased the risk of local-regional recurrence (OR = 2.272, 95% CI 1.734 to 2.977) and distant metastasis (OR = 1.777, 95% CI 1.369 to 2.307). Patients >65 years increased the risk of distant metastasis (OR 1.449, 95% CI 1.093 to 1.922), but reduced the risk of peritoneum implanting (OR 0.619, 95% CI 0.403 to 0.984). Patients with factors, including female (OR 1.687, 95% CI 1.164 to 2.444), signet ring cell cancer (OR 2.627, 95% CI 1.449 to 4.761) were at increased risk of peritoneum implanting. Receiving adjuvant chemotherapy ≤3 cycles could improve the risk of distant metastasis (OR 0.640, 95% CI 0.433 to 0.943). Receiving adjuvant radiotherapy could reduce the risk of distant metastasis (OR = 0.260, 95% CI 0.083 to 0.816), and potential reduced the risk of local-regional recurrence (OR = 0.302, 95% CI 0.085 to 1.078).

**Table 3:** The univariate and multivariate analyses of each recurrence pattern

	Local-regional recurrence				Distant metastasis				Peritoneum implanting			
Clinical features	Univariate analysis		Multivariate analysis		Univariate analysis		Multivariate analysis		Univariate analysis		Multivariate analysis	
	OR (95% CI)	*p* value	OR (95% CI)	*p* value	OR (95% CI)	*p* value	OR (95% CI)	*p* value	OR (95% CI)	*p* value	OR (95% CI)	*p* value
Age												
≦65												
>65	1.173 (0.908–1.516)	0.223	0.744 (0.537–1.030)	0.075	1.286 (1.008–1.641)	0.043	1.449 (1.093–1.922)	0.010	0.552 (0.370–0.822)	0.003	0.619 (0.403–0.984)	0.029
Sex												
Male												
Female	0.666 (0.502–0.882)	0.005	NA	NA	0.825 (0.639–1.065)	0.140	NA	NA	1.861 (1.330–2.603)	<0.001	1.687 (1.164–2.444)	0.006
Hospital												
Specialist hospitals												
General hospitals	1.964 (1.545–2.497)	<0.001	1.724 (1.312–2.265)	<0.001	1.664 (1.332–2.079)	<0.001	1.496 (1.164–1.940)	0.002	1.256 (0.910–1.733)	0.165	NA	NA
Tumor location												
Non-GEJ												
GEJ	1.780 (1.398–2.266)	<0.001	1.637 (1.236–2.165)	0.001	1.226 (0.973–1.546)	0.084	NA	NA	0.619 (0.431–0.890)	0.010	0.649 (0.437–0.965)	0.033
AJCC stage												
I–II												
III	1.455 (1.145–1.850)	0.002	NA	NA	2.364 (1.879–2.974)	<0.001	NA	NA	1.627 (1.165–2.272)	0.004	NA	NA
T stage												
T1–2												
T3–4	1.752 (1.297–2.368)	<0.001	1.495 (1.058–2.113)	0.023	2.230 (1.684–2.953)	<0.001	1.674 (1.210–2.316)	0.002	3.291 (1.934–5.599)	<0.001	3.427 (1.956–6.003)	<0.001
N stage												
N0												
N1–3	1.913 (1.430–2.558)	<0.001	NA	NA	3.179 (2.400–4.212)	<0.001	2.354 (1.659–3.341)	<0.001	1.808 (1.195–2.736)	0.005	NA	NA
Positive LN ratio												
≦0.33												
>0.33	2.287 (1.799–2.909)	<0.001	1.953 (1.493–2.551)	<0.001	2.512 (1.998–3.181)	<0.001	1.582 (1.214–2.128)	0.001	1.509 (1.097–2.078)	0.012	NA	NA
No. of LN dissection												
<15												
≧15	0.422 (0.332–0.537)	<0.001	NA	NA	0.629 (0.502–0.787)	<0.001	NA	NA	1.094 (0.794–1.507)	0.583	NA	NA
Histological type												
Well-differentiated tumors												
Poorly differentiated tumors	0.881 (0.666–1.166)	0.376	NA	NA	0.811 (0.623–1.057)	0.121	NA	NA	1.589 (1.022–2.472)	0.040	1.431 (0.889–2.303)	0.140
Signet ring cell cancer	1.308 (0.867–1.823)	0.201	NA	NA	0.961 (0.645–1.433)	0.846	NA	NA	3.576 (2.074–6.165)	<0.001	2.627 (1.449–4.761)	0.001
Lymphadenectomy extent												
D2												
D0/D1	2.442 (1.912–3.119)	<0.001	2.272 (1.734–2.977)	<0.001	1.595 (1.274–1.996)	<0.001	1.777 (1.369–2.307)	<0.001	0.933 (0.677–1.286)	0.673	NA	NA
Neo-adjuvant chemotherapy												
Yes												
No	1.285 (0.727–2.272)	0.338	NA	NA	1.052 (0.630–1.755)	0.847	NA	NA	1.560 (0.662–3.675)	0.309	NA	NA
Adjuvant chemotherapy												
No												
Yes	0.829 (0.598–1.149)	0.261	NA	NA	0.909 (0.665–1.242)	0.550	NA	NA	1.448 (0.883–2.376)	0.142	NA	NA
Adjuvant chemotherapy regimen												
No												
Fluorouracil-based regimens	0.801 (0.577–1.112)	0.185	NA	NA	0.883 (0.645–1.208)	0.436	NA	NA	1.417 (0.862–2.329)	0.169	NA	NA
Other regimens	1.649 (0.850–3.199)	0.139	NA	NA	1.677 (0.862–3.263)	0.128	NA	NA	1.867 (0.762–4.570)	0.172	NA	NA
Adjuvant chemotherapy cycles												
No												
≦3 cycles	0.754 (0.519–1.095)	0.138	NA	NA	0.707 (0.495–1.008)	0.056	0.640 (0.433–0.943)	0.024	1.222 (0.701–2.130)	0.479	NA	NA
>3 cycles	0.880 (0.626–1.237)	0.463	NA	NA	1.055 (0.762–1.461)	0.875	0.842 (0.588–1.206)	0.348	1.590 (0.956–2.646)	0.074	NA	NA
Adjuvant radiotherapy												
Yes												
No	2.609 (0.893–7.623)	0.080	3.316 (0.928–11.848)	0.065	2.184 (0.911–5.233)	0.080	3.846 (1.226–12.068)	0.021	1.229 (0.365–4.138)	0.740	NA	NA

### The analysis for recurrence timing

During the follow-up, 793 patients experienced recurrence with the overall median RFS of 29.00 months (IQR 12.07, 147.23). There were 554 patients suffering early recurrence, with the median RFS of 10.77 months (IQR 6.00, 15.83). Another 239 patients experienced late recurrence, with the median DFS of 35.83 months (IQR 28.67, 50.67). Patients with T3–4 (OR = 2.148, 95% CI 1.537 to 3.001), N1–3 (OR = 1.874, 95% CI 1.320 to 2.660), and LN+%≧0.33(OR = 2.024, 95% CI 1.531 to 2.681) contributed to the higher early recurrence risk. Having operation in general hospitals has potential to suffer early recurrence (OR = 1.292, 95% CI 0.995 to 1.676). Having lymph nodes resection no less than 15 (OR = 0.625, 95% CI 0.480 to 0.815) could reduce the risk of early recurrence.

**Fig. 3:**
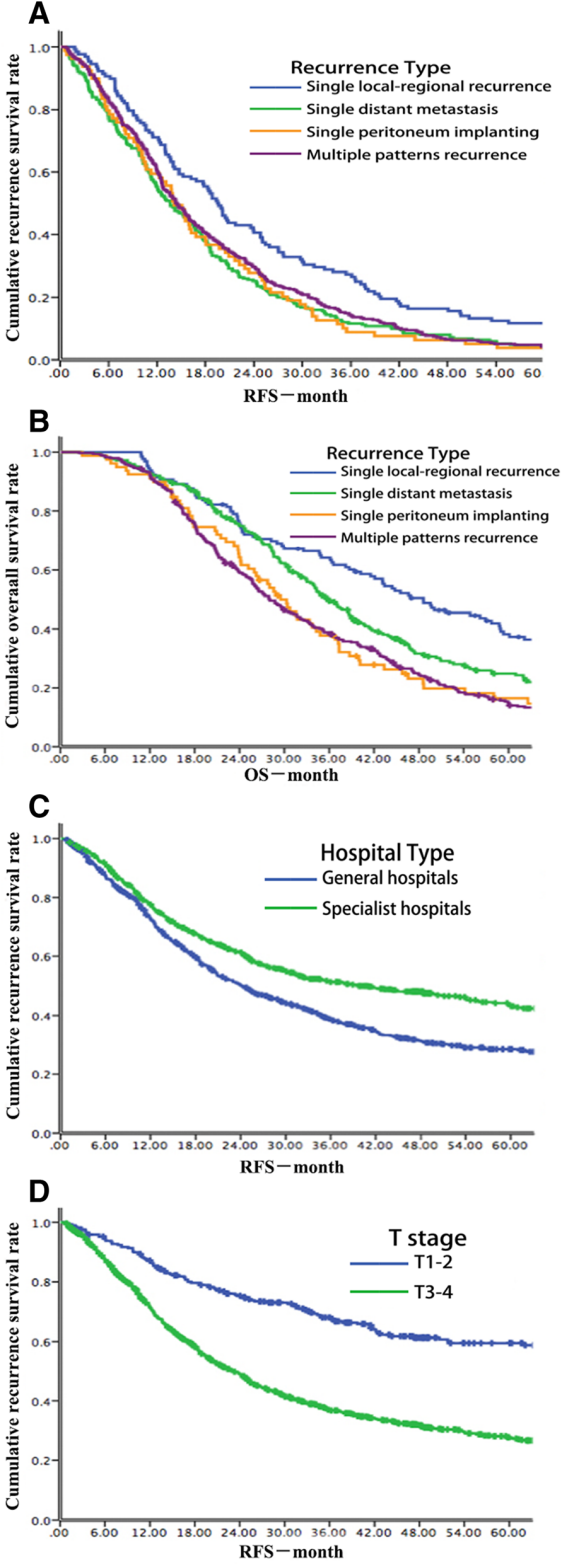
**a** The survival curves for RFS of different recurrence patterns. **b** The survival curves for OS of different recurrence patterns. **c** The survival curves for RFS of different surgical hospitals. **d** The survival curves for RFS of different T status

Among the recurrence patients, the median RFS was only 14.73 months (IQR 7.90, 26.17), and the median RFS varied from recurrence types. The median RFS of single local-regional recurrence, single distant metastasis, single peritoneum implanting, and multiple patterns recurrence was 19.47 months (IQR 10.40, 36.57), 13.67 months (IQR 6.90, 24.27), 14.17 months (IQR 7.73,25.33), and 14.33 months (IQR 7.80, 25.70), respectively (Fig. 3a).

### The survival outcome of each recurrence type

There were 668 patients dead during the follow-up, with overall survival of 50.94 months (IQR 26.53, 205.73). Among recurrent patients, the overall survival was as short as 33.63 months (IQR 20.63, 54.87). The median overall survival of single local-regional recurrence, single distant metastasis, single peritoneum implanting, and multiple patterns recurrence was 48.63 months (IQR 24.17, 122.40), 35.70 months (IQR 24.00, 57.80), 29.50 months (IQR 17.90,45.77), and 28.03 months (IQR 18.10, 47.37), respectively (Fig. 3b).

## Discussion

Most studies had noted that curative resection for gastric cancer focused largely on prognosis [15, 16]. In China, most recurrence pattern data was based on small sample, single-center database [10–12], which could not actually reflect the presence of gastric cancer treatment. And little studies noted recurrence timing and took the surgical hospitals’ influence on recurrence into consideration. This study was important, because it firstly aimed to identify the incidence, patterns, and timing of recurrence after curative resection for gastric cancer in a large sample and multiple-center cohort of Chinese patients. The outcomes not only provided Chinese presence of curative resection for gastric cancer but also informed the points, which clinicians should focus during the postoperative follow-up.

As this analysis noted, 60.8% patients experienced recurrence after curative resection for gastric cancer, which was similar with previous results [10, 12, 15]. The incidence of distant metastasis was comparably high (30–45%) all over the world [3, 5, 7, 8, 10–12, 17, 18]. Patients in Japan and South Korea suffered lower local-regional recurrence compared with China (7-10 vs. 32.4%) [7–9]. But American patients suffered higher local-regional recurrence in contrast with the Chinese (44.1 vs. 32.4%) [3]. However, peritoneum implanting in China was obviously lower than in Japan, South Korea, and USA (13.7 vs. 30–45.9%) [3, 7, 8, 18, 19]. It might associate with less sensitive imaging examination (even MRI/CT with only 56% sensitivity for peritoneum implanting) [20], less laparoscopic exploration, and cytological examination of peritoneal lavage fluid. The actual incidence of peritoneum implanting would be higher in China.

Based on this analysis, the difference between surgical hospitals and recurrence was specifically analyzed. Receiving operation in general hospitals contributed to higher recurrence risk and shorter RFS (Fig. 3c, *p* < 0.001). It partly related less lymph nodes harvest and lower D2 lymphadenectomy ratio in general hospital. Although the time span of this study was as long as 27 years and D2 lymphadenectomy was just generalized in the past 15 years, specialist hospitals had higher lymph nodes harvest ≧15 rate D2 lymphadenectomy rate at every period (from Jan. 1986 to Dec. 1999, 40.7 and 34.0% vs. 19.5 and 13.3%; from Jan. 2000 to Dec. 2006, 69.0 and 58.1% vs. 33.2 and 29.9%; from Jan. 2007 to Sept. 2013, 77.3 and 73.6% vs. 59.1 and 57.4%). It might relate to less volume of curative gastric cancer resection in general hospitals [21] and earlier D2 lymphadenectomy generalization in specialist hospitals. It also referred that standard D2 lymphadenectomy training of clinicians should be continued in China. And increased T stage was the most important independently risk factor of recurrence and with worse RFS (Fig. 3d, *p* < 0.001). Several studies had noted that postoperative recurrence associated with factors, such as T stage, extent of lymph node invasion, and tumor location [3, 7, 10–12, 17, 19, 22–26], which were consistent with this study. Based on this database, female and signet ring cell cancer contributed to increased incidence of peritoneum implanting, which reported in previous results [17, 27]. However, we did not find that lymph node invasion related to peritoneum implanting [22]. The age of patients closely associated with distant metastasis and peritoneum implanting. Patients older than 65 years old had higher risk of distant metastasis and lower risk of peritoneum implanting. In further study, in the group of patients older than 65 years old, less patients were signet ring cell cancer (5.9 vs. 13.0%, *p* < 0.001) and more patients did not receive adjuvant chemotherapy (22.6 vs. 12.9%, *p* < 0.001), comparing with the group of patients no more than 65 years old. It might explain the results. In addition, among patients with signet ring cell cancer, compared with subtotal gastrectomy, patients with total gastrectomy contribute to slightly lower risk of peritoneum implanting (10.0 vs. 15.2%) and multiple patterns recurrence (23.3 vs. 33.0%). It might be better to underwent total gastrectomy for signet ring cell cancer patients.

The overall median RFS noting in this analysis was 29.00 months, which was similar to the results in USA [3]. Of note was the further study that the median RFS among patients with recurrence was much shorter, at a little more than 1 year (14.73 months), as the previous study reported [17, 19]. Single local-regional recurrence occurred later than any other recurrence types and had better overall survival. The overall 3- and 5-year survival rates were 63.8 and 44.8%, respectively, which were higher than previous American study (50.9 and 39.3%) [3]. In further study, the median survival after recurrence was only 13.97 months (IQR 7.03, 24.67), which was consistent with the results of Koizumi W and colleague (13.0 months) [28]. And early recurrence patients had worse survival after recurrence than late recurrence patients (13.50 vs. 16.30 months, *p* = 0.023), which did not show in American analysis [3].

In this study, Chinese patients had more multiple pattern recurrence than patients in America and South Korea (42.5 vs. 33.2 vs. 16.3–27.4%) [3, 19, 29]. It partly reflected the lack of regular postoperative follow-up in China. A large part of recurrence was early recurrence and 5-year DFS was only 5.2%. So, that receiving operative examination every 3 months within 2 years and every 6 months within 5 years after surgery were recommended [30, 31], especially for patients with such risk factors.

There were also several limitations in this study. At first, as the patients undergoing surgery in more than 100 hospitals in China, selection bias was unavoidable. Nevertheless, in China, it was general for patients receiving radical resection and adjuvant treatment in different hospitals. Secondly, several other factors previously reported as associated with the recurrence, such as tumor size, vascular tumor thrombus, and Lauren classification did not include this study. Thirdly, the relationship of D2 lymphadenectomy and lymph nodes harvest ≧15 to recurrence were different, which associated with long time span of surgery and loss of detail surgical records. And then, in this study, we only found had no more than 3 cycles of adjuvant chemotherapy could reduce the recurrence, with statistical significance. More cycles of adjuvant chemotherapy also potential to reduce recurrence. It might associate with different chemotherapy regimens and bias in retrospective study. However, we could not find the association between neo-adjuvant chemotherapy, adjuvant chemotherapy regimens, and recurrence. And although we found that adjuvant radiotherapy could reduce postoperative recurrence and distant metastasis, the results were lower reliability because of small samples. Last but not the least, some patients were lost during follow-up, without information on recurrence or death. All these issues might have led to potential bias in the analysis of recurrence patterns and timing after curative gastric cancer resection.

## Conclusions

In brief, this multiple-center retrospective analysis noted that postoperative recurrence of gastric cancer was common, especially early recurrence. Advanced tumor stage and large tumor burden contributed to increased recurrence risk and worse survival. Enough lymph nodes harvest and standard D2 lymphadenectomy could reduce the postoperative recurrence. Patients might benefit from adjuvant chemotherapy and radiotherapy in China.

## Abbreviations

AJCC: American Joint Committee on Cancer
CI: Confidence internal
DFS: Disease-free survival
GEJ: Gastroesophageal junction
IQR: Interquartile range
OR: Odd ratio
OS: Overall survival
